# Entomological drivers of uneven malaria transmission in urban lowland areas in Bouaké, Côte d’Ivoire

**DOI:** 10.1186/s12936-023-04457-x

**Published:** 2023-01-31

**Authors:** Milossé M. C. Dahoui, Kouassi A. Adou, Baba Coulibaly, Koffi L. Niamien, Aboubacar Koné, Sylvie Cornelie, Dounin D. Zoh, Konan F. Assouho, Nicolas Moiroux, Akré M. Adja, Florence Fournet

**Affiliations:** 1 MIVEGEC (Université de Montpellier, CNRS, IRD), 911 Avenue Agropolis, BP 64501, 34394 Montpellier, France; 2 Institut Pierre Richet, Institut National de Santé Publique, 01 BP 1500, Bouaké, Côte d’Ivoire; 3 Unité de Formation et de Recherche Biosciences, Université Félix Houphouët-Boigny, 08 BP 3800, Abidjan, Côte d’Ivoire; 4 Centre d’Entomologie Médicale et Vétérinaire, BP V 18 01, Bouaké, Côte d’Ivoire

**Keywords:** Urban agriculture, Lowlands, *Anopheles*, Malaria risk, Côte d’Ivoire

## Abstract

**Background:**

The use of urban lowlands for agriculture contributes to the food security of city- dwellers, but promotes malaria transmission. The objective of the study was to characterize the entomological drivers of malaria transmission in two lowlands (N’Gattakro and Odiennekourani) in the city of Bouaké, Côte d’Ivoire.

**Methods:**

The human landing catch technique was used to capture mosquitoes in houses located at the edge of two lowlands in Bouaké from February to December 2019. Cultivated surfaces were calculated monthly in both lowlands for each crop type (rice and market gardening) using images acquired by a drone. The different mosquito species were identified morphologically and by PCR analysis for the *Anopheles gambiae* complex. *Anopheles* infection by *Plasmodium* parasites was assessed by quantitative PCR. Mosquito diversity, biting behaviour and rhythmicity, and malaria transmission were determined in each lowland and compared.

**Results:**

*Anopheles gambiae *sensu lato (*s.l.*) was predominant in N’Gattakro and *Culex quinquefasciatus* in Odiennekourani. Four *Anopheles* species were identified: *An. gambiae s.l.* and *Anopheles funestus s.l.* in both lowlands, *Anopheles pharoensis* in N’Gattakro, and *Anopheles ziemanni* in Odiennekourani. Within the *An. gambiae* complex, three species were caught: *An. gambiae *sensu stricto (s.s.), *Anopheles coluzzii*, and *Anopheles arabiensis* for the first time in Côte d’Ivoire (30.1%, 69.9% and 0% in N’Gattakro, and 45.1%, 52.6% and 2.4% in Odiennekourani, respectively). *Anopheles gambiae s.l.* species exhibited a significant exophagic behaviour in N’Gattakro (77.1% of outdoor bites versus 52.2% in Odiennekourani). In N’Gattakro, 12.6% of captures occurred before bedtime (09.00 pm) and after waking up (05.00 am), 15.1% in Odiennekourani. The mean human biting rate was higher in N’Gattakro than in Odiennekourani (61.6 versus 15.5 bites per person per night). Overall, *Anopheles* infection rate was 0.68%, with 0.539 and 0.029 infected bites per person per night in N’Gattakro and Odiennekourani, respectively.

**Conclusion:**

The risk of malaria in urban agricultural lowland areas is uneven. The role of agricultural developments and irrigation patterns in the production of larval habitat should be explored. The exophagic behaviour of *Anopheles* vectors raises the question of the residual transmission that needs to be assessed to implement appropriate control strategies.

## Background

Malaria is the most prevalent and deadly vector-borne disease in the world. In 2020, the estimated number of malaria cases was 241 million worldwide of which 95% occurred in Africa populations [1]. In the same year, 627,000 malaria-related deaths were estimated with children under 5 facing the most significant burden among [1]. In Côte d’Ivoire, 7,571,801 malaria cases and 15,913 malaria deaths were reported in 2020 [1]. The overall incidence rate of malaria in Côte d’Ivoire was 230‰ in 2019 [2]. Malaria is endemic with 81% of population living in areas with an annual incidence ranging from 300 to over 500‰ [2]. The objectives of the National Strategic Plan for 2021–2025 are to reduce malaria mortality rates and incidence of malaria cases by at least 75% compared to 2015 [2].

Although malaria has long been considered a rural disease, it is now also present in cities [3]. Urban malaria is a very serious challenge because 50% of the world population is already living in cities and this percentage is expected to increase to 70% in 2050 [4]. In Côte d’Ivoire, 51.2% of the population currently lives in urban areas [5]. Moreover, population concentration in cities raises the issue of food supply, and more broadly of food security [6]. Urban agriculture has emerged as a promising opportunity to enhance food security [7]. It also contributes to the local economic development, poverty reduction and social inclusion of city dwellers as well to greening the city, urban waste recycling, and reducing vulnerability to climate change.

In sub-Saharan African cities, lowlands are privileged areas for market gardening and rice growing [8]. In Côte d’Ivoire, they have been used for agricultural purposes for decades [9]. However, they also constitute areas conducive to malaria vector development by offering permanent larval habitats [10–12].

The aim of this study was to assess malaria transmission in the neighbourhood of two lowlands in relation to their agricultural development practices in the city of Bouaké, Côte d’Ivoire.

## Methods

### Study site

The study was conducted in the city of Bouaké that is located in the centre of Côte d’Ivoire, approximately 350 km for Abidjan, the capital city. According to the most recent census of 2014 [13], Bouaké has 536,719 inhabitants.

Annual malaria incidence in Bouaké was estimated at 400–499‰ in the southern health district and over 500‰ in the north-eastern and north-western health districts in 2018 [14]. In Bouaké, malaria prevention is mainly achieved through the distribution of long-lasting insecticidal nets (LLINs) to the entire population. The utilization rate of LLINs increased from 50% in 2016 to 63.2% in 2019 [15]. There are no indoor residual spraying (IRS) or larvicide campaigns, which are reserved for a few pilot areas in the country. The population normally receives health care advice on how to clean up their environment.

**Fig. 1:**
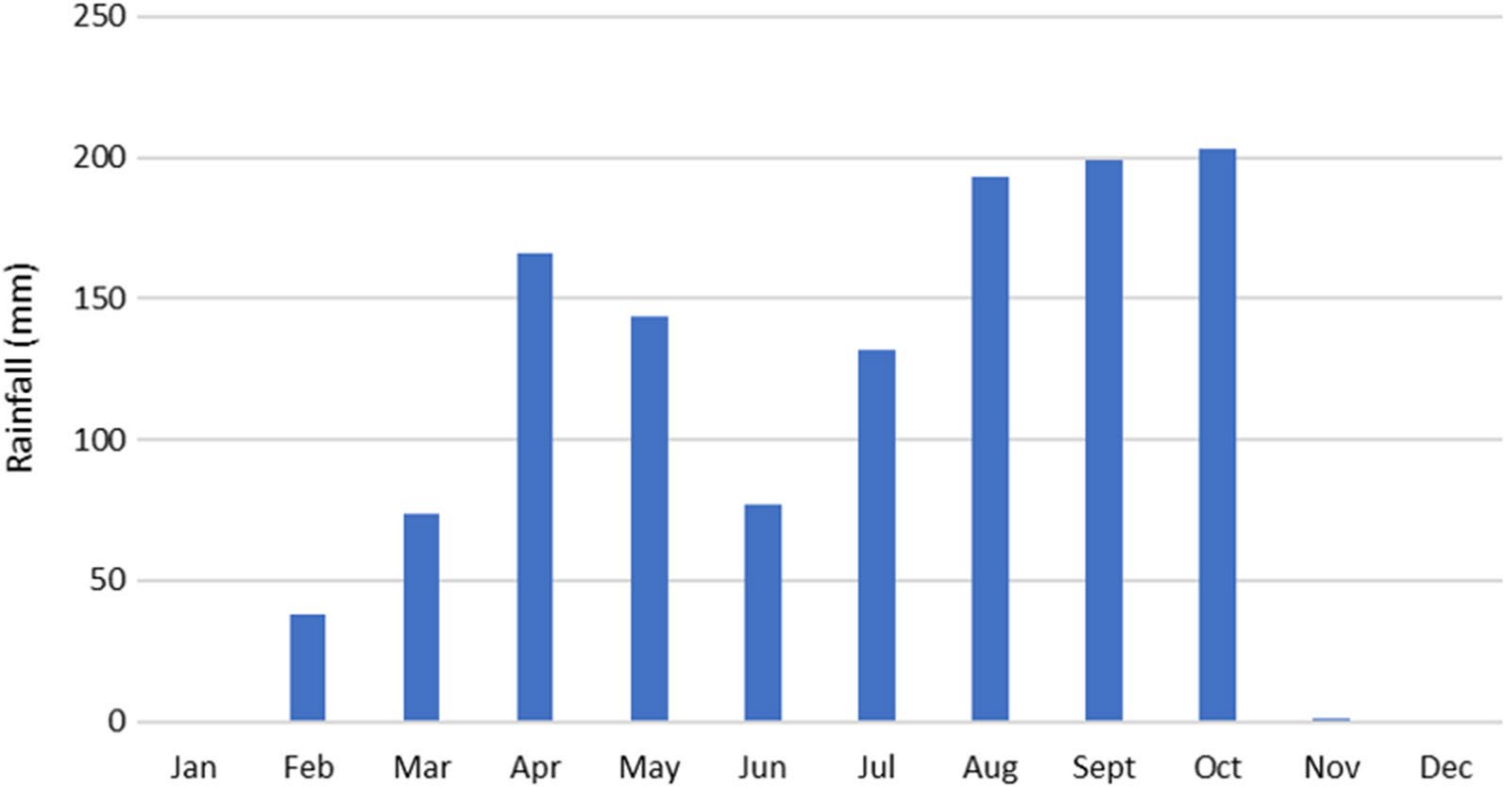
Monthly variation of the amount of rainfall in Bouaké in 2019 (source: SODEXAM, 2022)

The climate is tropical humid with two seasons: a rainy season between April and October, and a dry season from November to March [16]. The monthly average temperatures range between 24 and 28 °C and the annual rainfall between 1000 and 1200 mm [17]. According to the national society of airports and meteorology (SODEXAM) estimates, the rainfall total was 1227 mm in 2019 (Fig. 1).

**Fig. 2:**
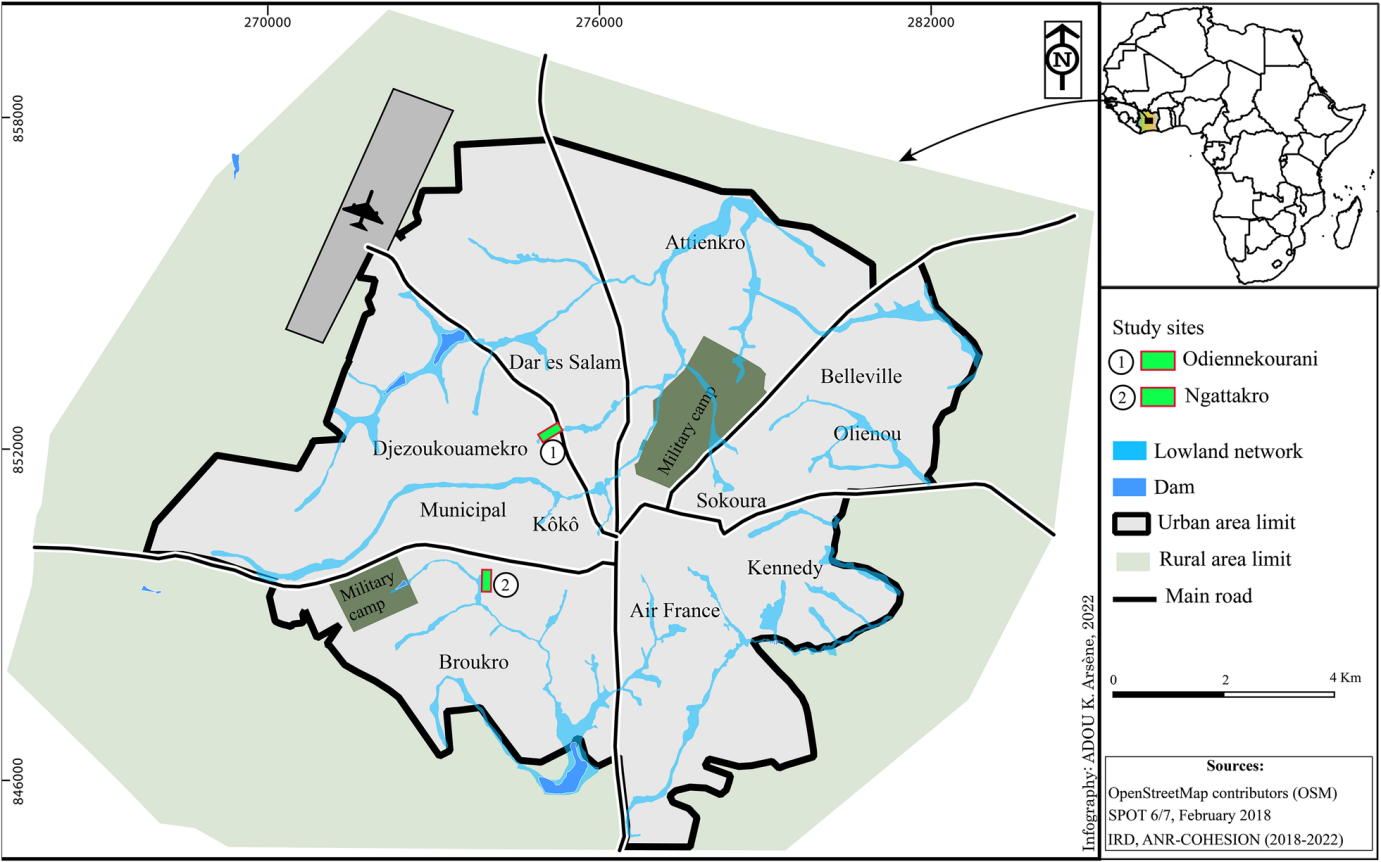
Map of Bouaké with the location of the study sites

The city covers approximately 10,000 ha crossed by a large hydrographic network [9] (Fig. 2). This creates many lowlands that split the city and account for more than 1200 ha (i.e. ~ 12% of the total city area). These lowlands are intensively exploited for market gardening and rice cultivation.

**Fig. 3:**
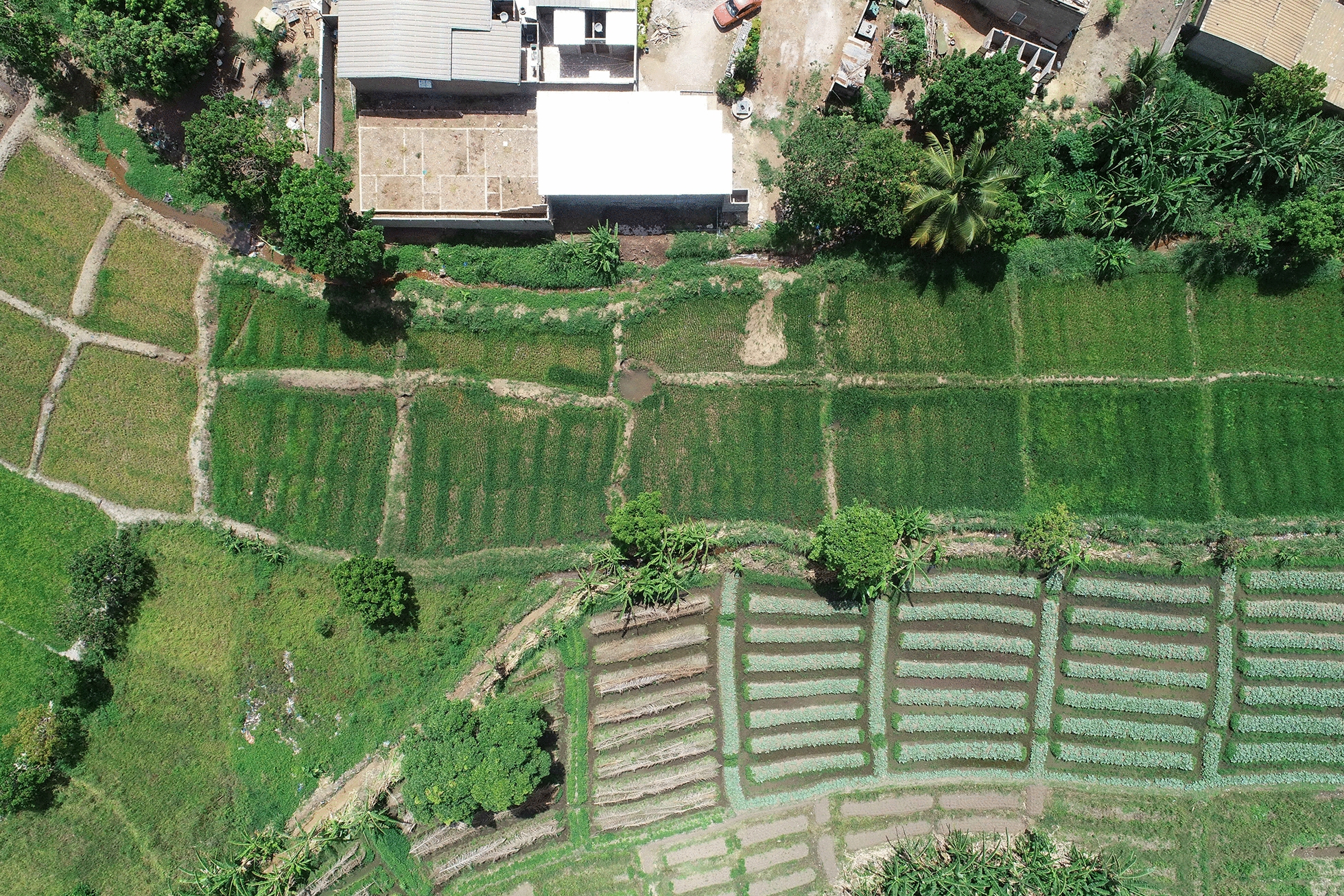
Aerial view of N’Gattakro lowland showing market gardening plots (at the bottom) and rice fields (left and upper part) with irrigation channels. The house corresponds to the second mosquito sampling site (April 2019)

**Fig. 4:**
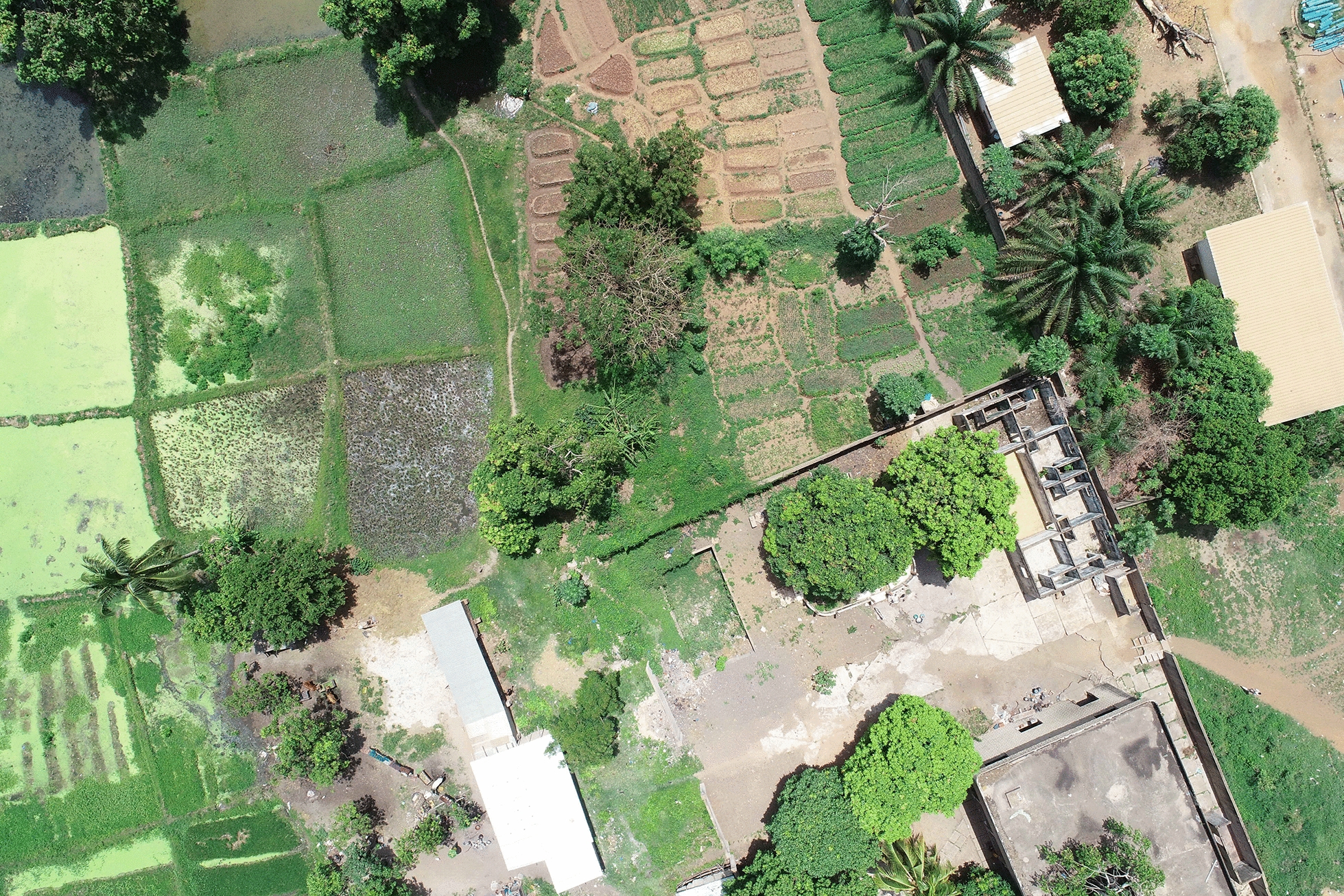
Aerial view of Odiennekourani lowland showing irrigated rice fields (left) and market gardening plots (right). The closed compound in the lower right corner is the second mosquito sampling site (April 2019)

For this study, two lowlands were selected. These two lowlands are quite similar in size and cropping patterns. The first one is in the N’Gattakro district, a structured district from the 1970s, located in the southwest part of the city, and covers 2.5 ha (Fig. 3). The farmers do not live close to the lowlands which are part of the Kan river basin. The second has a surface area of 2.8 ha and is located in the Odiennekourani district, an old district with a high building density in the surrounding area, located in the northwest part of the city (Fig. 4). This lowland is part of the Loka river basin and it is farmed by local residents. In these lowland, rice and market-garden crops (cabbage, okra, carrots, onions, lettuce, eggplants, corn) are grown. Unlike N’Gattakro, market gardening plots in Odiennekourani are watered manually. Rice plots are permanently irrigated in N’Gattakro by a watercourse, whereas the stream circulation can be interrupted in Odiennekourani. In addition, in Odiennekourani, the neighbourhood wastewater is discharged resulting in poor water quality.

### Environmental data

The agricultural development of the two lowlands was monitored by monthly image acquisition with a drone (DJI Phantom 4 Pro V2) between January and December 2019. The drone flight parameters (flight height: 75 m, flight speed: 5.2 m/second, image capture every 7 s, spatial resolution: 2 cm/pixel, angle of view: 90° relative to the ground, percentage of overlap (front and side): 70%) were defined automatically and kept for each flight. The monthly images were assembled into a single georeferenced image using the OpenDroneMap platform (https://www.opendronemap.org/). Each lowland was divided into homogeneous spatial units, according to the agricultural use (rice cultivation, market gardening) by photo-interpretation with the free GIS software QGIS (version 3.16.4). This analysis gave 67 homogeneous spatial units in the Odiennekourani lowland and 85 in the N’Gattakro lowland. Each parcel was assigned an ID. A field survey was carried out monthly to assign each spatial unit a crop use (“rice” or “market gardening”) and to calculate the corresponding area.

### Mosquito collection

Adult mosquitoes were caught by the human landing catching (HLC) method that is still considered the “gold standard” to measure *Anopheles* vector biting on humans [18], in inhabited areas located along the two lowlands from February to December 2019 (except in January, April and May due to human resource constraints). For comparison with a recent study in Bouaké [11], the entomological data were organized by season: dry season (February, March, November and December) and rainy season (June to October).

**Fig. 5:**
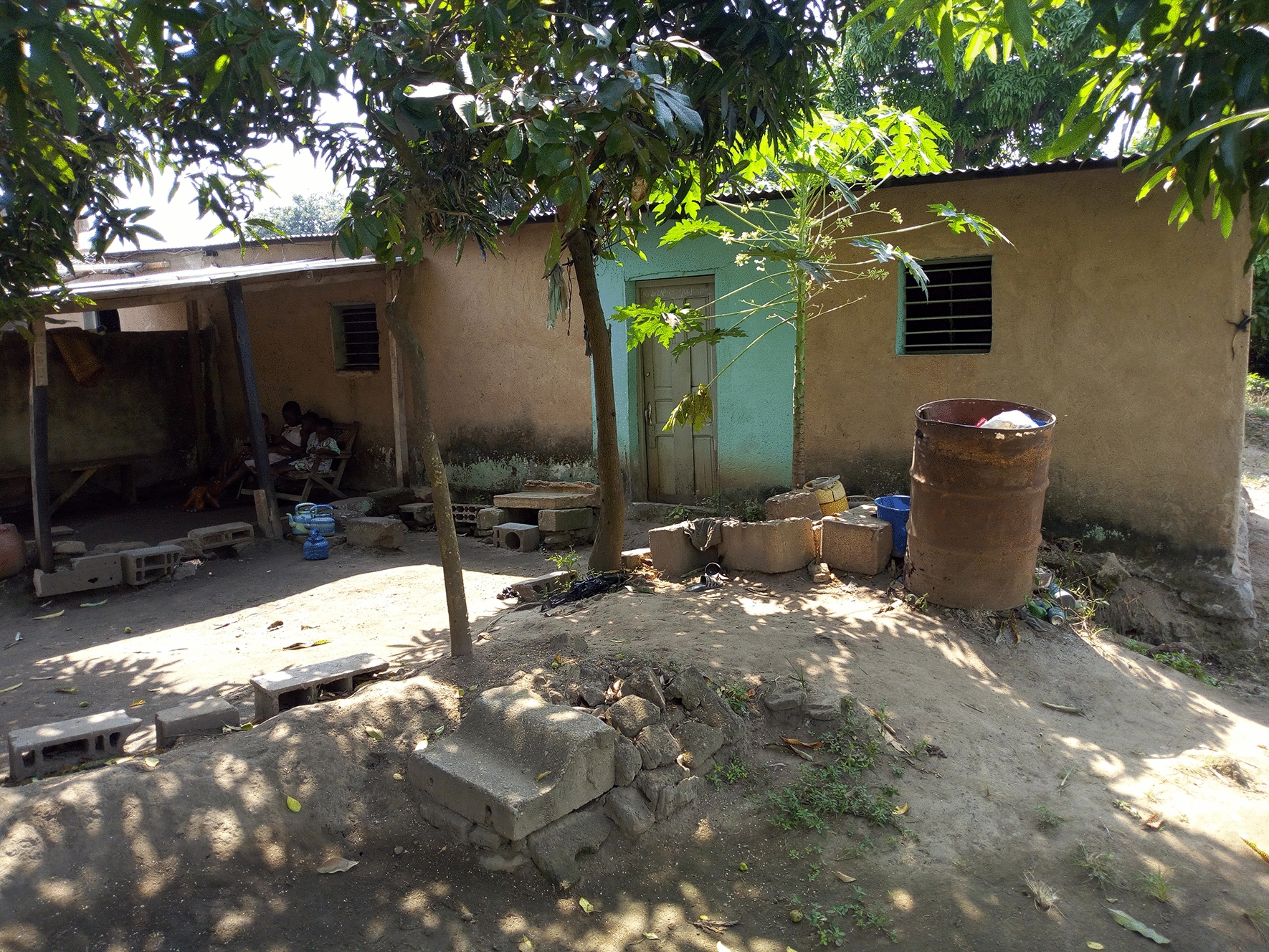
A mosquito catching house located close to the lowland in the Odiennekourani district

Mosquitoes were collected inside and outside two houses (Fig. 5) in each area (four person-nights per neighbourhood per month) from 07:00 pm to 07:00 am during one night per lowland and per month (for a total of 72 nights of HLC sampling). Houses were selected based on the agreement of their inhabitants and the same houses were used throughout the study. The mean distance between the house and the lowland was 26 m meters in N’Gattakro and 19 m in Odiennekourani. Mosquitoes were morphologically identified to species or complex level according to Mattingly [19] and Gilles & Coetzee [20]. Only *Anopheles* specimens were individually preserved in 1.5 ml Eppendorf tubes containing silica gel for further analysis.

### Molecular analyses

DNA was extracted from the head and thorax following the method described by Yahouedo et al. [21]. Twin species of *Anopheles gambiae *sensu lato (*s.l.)* were identified by PCR according to Favia et al. [22] to distinguish *Anopheles coluzzii* and *An. gambiae *sensu stricto (s.s.) [23]. *Anopheles arabiensis* and *An. gambiae s.l.* were differentiated according to Scott et al. [24].

For the molecular identification of *An. gambiae s.l.* species, because of high densities in February, March, and September, only 56.4% and 92.6% of the *Anopheles* collected in N’Gattakro and in Odiennekourani, respectively, were analysed after random selection.

The presence of *Plasmodium* parasites was assessed by quantitative PCR (qPCR) based on Mangold et al. [25] in the subsample of *An. gambiae* complex specimens identified by molecular analysis and in all individuals of the other species (*Anopheles funestus s.l., Anopheles ziemanni, Anopheles pharoensis*).

### Entomological parameters measured

The human biting rate (HBR) was derived from the number of host-seeking female mosquitoes landing on subjects. The HBR was calculated as the number of all female anopheles collected per person per night, which resulted in the number of bites per person per night (bpn) [26]; the infection rate (IR) as the proportion of *Anopheles* infected by *Plasmodium* parasites. The entomological inoculation rate (EIR) was calculated by multiplying the HBR by the IR, as the number of infected bites per person per night (ibpn) [27].

### Statistical analysis

Statistical analyses were performed with the R software version 4.1 [28], and a 5% significant threshold. The Spearman test was used to assess the correlation between rainfall and cultivated areas, and between rainfall and mosquito abundance [29]. The Chi^2^ test was used to compare the mosquito species abundance according to the lowland, the season and the collection position (indoors and outdoors) [30]. The Fisher’s exact test was used to compare the number of infected *Anopheles* specimens between lowlands [30]. The Wilcoxon-Mann Whitney test was used to compare the human biting rate and the entomological inoculation rate between lowlands [30].

## Results

### Agricultural development of lowlands

**Fig. 6:**
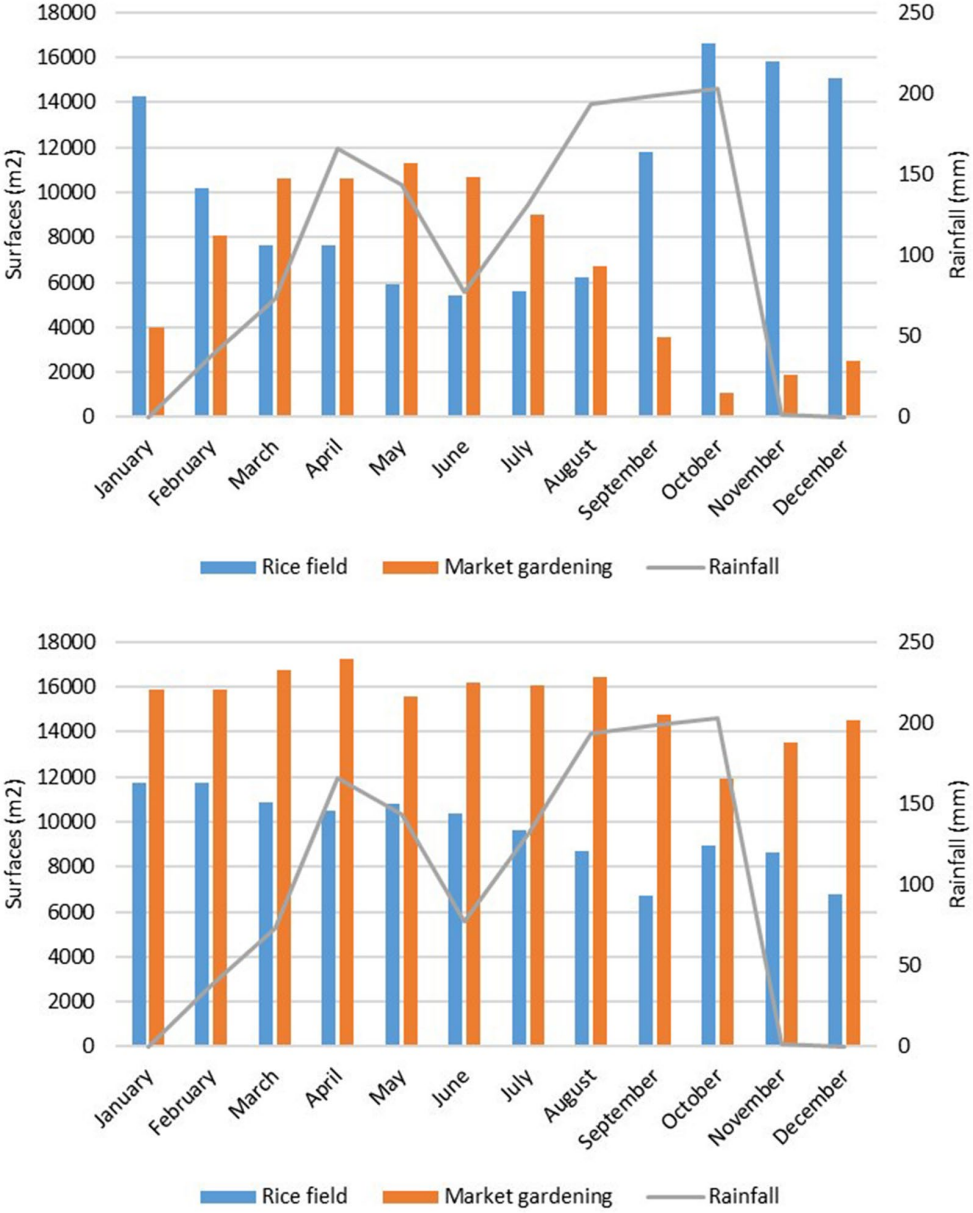
Monthly rainfall and agricultural utilization of the lowlands in N’Gattakro (top) and in Odiennekourani (bottom) in 2019

The area devoted to rice fields was ~ 10,000 m^2^ in both lowlands. On average, market gardening represented 6600 m^2^ in N’Gattakro and 15,400 m^2^ in Odiennekourani. In N’Gattakro, the market gardening surface varied over time, and was largest between March and June, when rainfall started to increase. It remained almost constant in Odiennekourani (Fig. 6). Similarly, rice culture surface increased in N’Gattakro from August to January with a maximum in October at the peak of rainfall. Conversely, it did not show any major variability in Odiennekourani. These variations were not linked to the rainfall pattern (*p* > *0.1*).

### Mosquito characterization

In total, 7270 female mosquitoes were collected. They belonged to four genera (*Aedes*, *Anopheles*, *Culex*, and *Mansonia*) and to 18 species (Table 1). Species composition differed between the two lowlands: 16 species in N’Gattakro (2 species of *Aedes*, 3 species of *Anopheles*, 9 species of *Culex*, 2 species of *Mansonia*) and 13 species in Odiennekourani (2 species of *Aedes*, 3 species of *Anopheles*, 6 species of *Culex*, and 2 species of *Mansonia*).

**Table 1:** Characterization of mosquito species in N'Gattakro and Odiennekourani

Species	N’Gattakro n (%)	Odiennekourani n (%)	Total n (%)
***Aedes***			
*Aedes aegypti*	5 (0.12)	5 (0.16)	10 (0.14)
*Aedes ochraceus*	1 (0.02)	0 (0)	1 (0.01)
*Aedes vittatus*	0 (0)	1 (0.03)	1 (0.01)
***Anopheles***			
*An. funestus s.l*	14 (0.34)	8 (0.25)	22 (0.3)
*An. gambiae s.l*	2198 (54.08)	544 (16.97)	2742 (37.71)
*An. pharoensis*	5 (0.12)	0 (0)	5 (0.07)
*An. ziemanni*	0 (0)	6 (0.19)	6 (0.08)
***Culex***			
*Culex annulioris*	1 (0.02)	0 (0)	1 (0.01)
*Culex antennatus*	102 (2.51)	96 (2.99)	198 (2.70)
*Culex decens*	65 (1.60)	8 (0.25)	73 (1.00)
*Culex gigateus*	5 (0.12)	0 (0)	5 (0.07)
*Culex ingrami*	126 (3.10)	103 (3.25)	229 (3.15)
*Culex moucheti*	257 (6.32)	455 (14.19)	712 (9.79)
*Culex nebulosus*	9 (0.22)	0 (0)	9 (0.12)
*Culex quinquefasciatus*	1228 (30.22)	1844 (57.52)	3072 (42.25)
*Culex tigripes*	1 (0.02)	2 (0.06)	3 (0.04)
***Mansonia***			
*Mansonia africana*	40 (0.98)	120 (3.74)	160 (2.20)
*Mansonia uniformis*	7 (0.17)	14 (0.44)	21 (0.28)
Total	4064	3206	7270

**Fig. 7:**
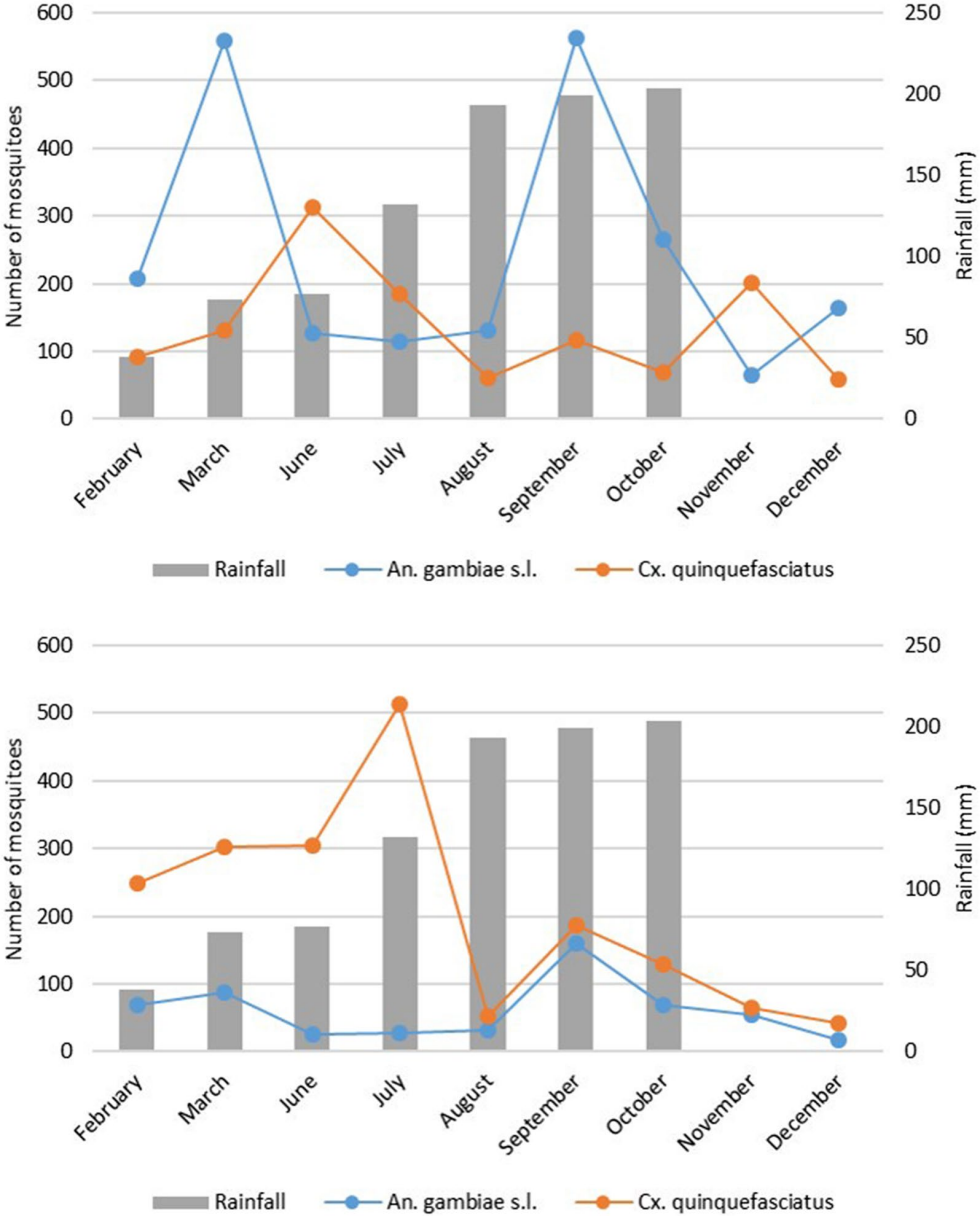
*An. gambiae s.l.* and *Cx. quinquefasciatus* abundance variations in N'Gattakro (top) and in Odiennekourani (bottom) in function of the rainfall during the study period

In N'Gattakro, *An. gambiae s.l.* was the predominant species representing 54.08% of captures, whereas *Culex quinquefasciatus* accounted for 30.22% of captures. In Odiennekourani, *Cx. quinquefasciatus* was dominant (57.52%), followed by *An. gambiae s.l.* representing 16.97% of the captures. These differences in abundance between lowlands were significant (Chi^2^ = 966.7; *p* < *0.0001*). Seasonal variations of abundance of *An. gambiae s.l.* followed a similar trend in both areas, with two peaks in March at rainfall onset, and in September just before the rainfall peak, but with different rates. Conversely, *Cx. quinquefasciatus* was mainly captured in June, July and November in N’Gattakro, and between February and July in Odiennekourani (Fig. 7). No correlation was found between rainfall and *Cx. quinquefasciatus* (*p* > *0.1*) or *An. gambiae s.l.* (*p* > *0.1*) abundances in both lowlands.

### Anopheles composition

*Anopheles gambiae s.l.* was represented more than 96% of the captures regardless of the lowland and the season (Table 2). *Anopheles funestus s.l.* was captured at low frequency in both lowlands and seasons. Few specimens of *An. pharoensis* (n = 5) and *An. ziemanni* (n = 6) were collected only in N’Gattakro and in Odiennekourani, respectively, and only during the rainy season.

**Table 2:** Seasonal distribution of *Anopheles* species in N'Gattakro and in Odiennekourani

Species	Dry season		Rainy season	
	n (%)		n (%)	
	N’Gattakro	Odiennekourani	N’Gattakro	Odiennekourani
*An. funestus s.l*	6 (0.60)	2 (0.86)	8 (0.66)	6 (1.85)
*An. gambiae s.l*	997 (99.40)	231 (99.14)	1.201 (98.93)	313 (96.31)
*An. pharoensis*	0 (0)	0 (0)	5 (0.41)	0 (0)
*An. ziemanni*	0 (0)	0 (0)	0 (0)	6 (1.85)
Total	1.003 (100)	233 (100)	1.214 (100)	325 (100)

*N* number of mosquitoes

### An. gambiae complex species

Among the 1740 *An. gambiae s.l.* analysed, 1129 (64.9%) were *An. coluzzii*, 599 (34.4%) were *An. gambiae *sensu stricto (s.s.), and 12 (0.7%) were *An. arabiensis* (Table 3). The distribution varied according to the lowland. *An. coluzzii* was significantly more frequent than *An. gambiae*s.s. in N’Gattakro compared to Odiennekourani in the rainy (Chi^2^ = 38.10; *p* < *0.0001*) and also dry season (Chi^2^ = 4.75; *p* = *0.03*). *Anopheles arabiensis* was found only at one site in Odiennekourani, mostly during the rainy season.

**Table 3:** Distribution of *An. gambiae s.l.* species identified by PCR analysis

	N’Gattakro n (%)	0diennekourani n (%)	Total n (%)
Dry season			
*An. arabiensis*	0 (0)	1 (0.5)	1 (0.2)
*An. coluzzii*	263 (64.1)	121 (55.0)	384 (61.0)
*An. gambiae s.s*	147 (35.9)	98 (44.5)	245 (38.9)
Rainy season			
*An. arabiensis*	0 (0)	11 (3.8)	11 (1.0)
*An. coluzzii*	600 (72.8)	145 (50.7)	745 (67.1)
*An. gambiae s.s*	224 (27.2)	130 (45.5)	354 (31.9)
All			
*An. arabiensis*	0 (0)	12 (2.4)	12 (0.67)
*An. coluzzii*	863 (69.9)	266 (52.6)	1,129 (64.9)
*An. gambiae s.s*	371 (30.1)	228 (45.1)	599 (34.4)
Total	1234	506 (100)	1740 (100)

### Anopheles gambiae complex biting behaviour

Species of the *An. gambiae* complex were more likely to be exophagic in N'Gattakro (77.1%, 951/1234) than in Odiennekourani (52.2%, 264/506). In N’Gattakro, significantly more *An. coluzzii* were collected outdoors than in Odiennekourani in the rainy (Chi^2^ = 13.4; *p* = *0.0002*) and dry season (Chi^2^ = 45.4; *p* < *0.0001*) (Table 4). It was the same for *An. gambiae s.s.* in the rainy (Chi^2^ = 19.28; *p* < *0.0001*) and dry season (Chi^2^ = 24.3; *p* < *0.0001*). The exophagy rate of *An. arabiensis* was 66.7%.

**Table 4:** Exophagy rate of *An. gambiae* complex species

			Outdoor n (%)	Indoor n (%)
N’Gattakro	Dry season	*An. arabiensis*	0 (0)	0 (0)
		*An. coluzzii*	203 (77.2)	60 (22.8)
		*An. gambiae s.s*	109 (74.1)	38(25.9)
	Rainy season	*An. arabiensis*	0 (0)	0 (0)
		*An. coluzzii*	465 (77.5)	135 (22.5)
		*An. gambiae s.s*	174 (77.7)	50 (22.3)
Odiennekourani	Dry season	*An. arabiensis*	1 (100)	0 (0)
		*An. coluzzii*	51 (42.1)	70 (57.9)
		*An. gambiae s.s*	42 (42.9)	56 (57.1)
	Rainy season	*An. arabiensis*	7 (63.6)	4 (36.4)
		*An. coluzzii*	91 (62.8)	54 (37.2)
		*An. gambiae s.s*	72 (55.4)	58 (44.6)

**Fig. 8:**
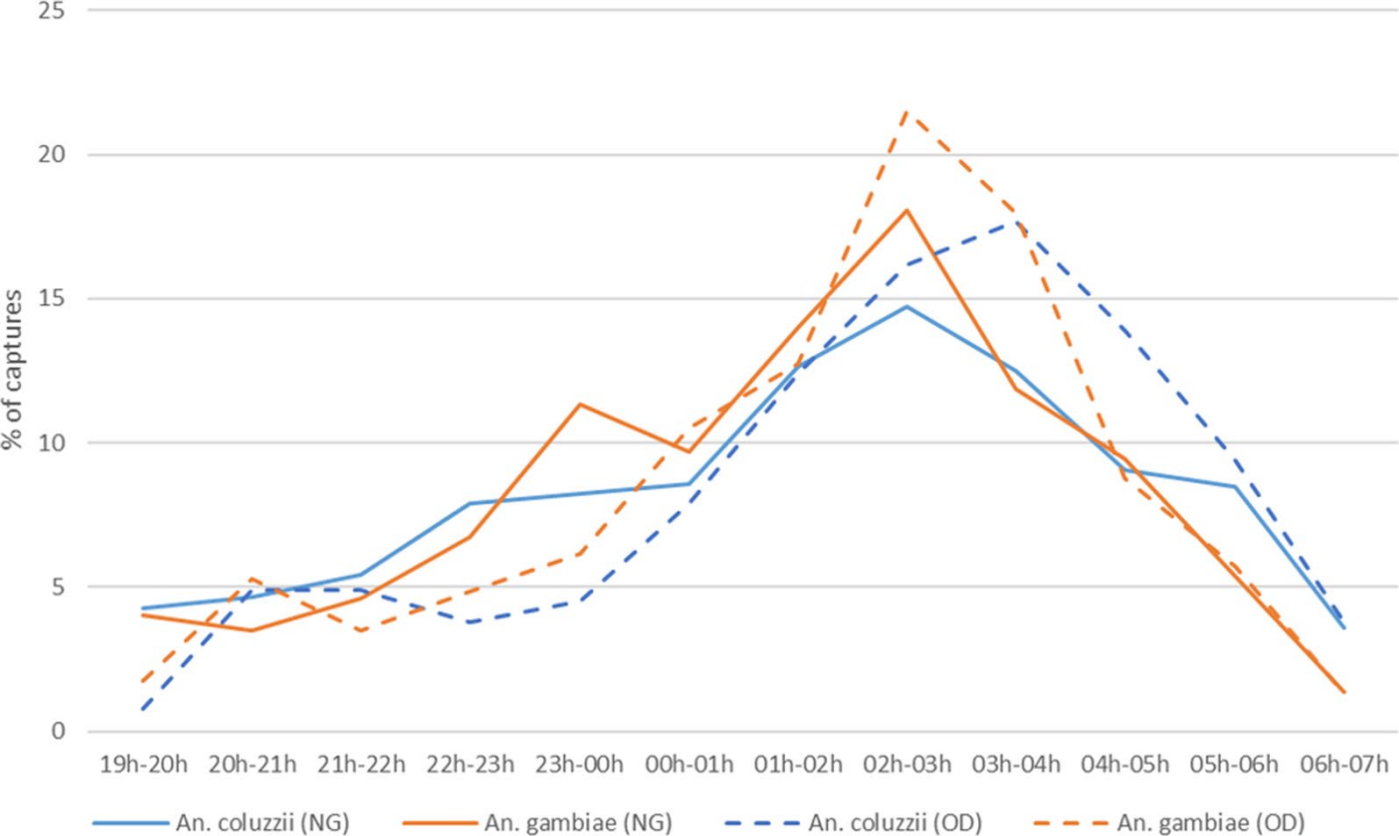
Mean hourly biting activity profiles of *An. gambiae s.s.* and *An. coluzzii* in N’Gattakro (NG) and in Odiennekourani (OD)

In N’Gattakro, the biting activity of *An. coluzzii* and *An. gambiae s.s.* peaked between 02:00 am and 03:00 am (Fig. 8). In Odiennekourani, the biting activity of *An. gambiae s.s.* followed the same pattern as in N'Gattakro, in contrast to *An. coluzzii* which exhibited a maximum activity between 03:00 and 04:00 am. Overall, 12.6% (in N’Gattakro) and 15.1% (in Odiennekourani) of *An. gambiae s.l.* were captured between 07:00 and 09:00 pm, and between 05:00 and 07:00 am.

### Plasmodium parasite infection and transmission

Overall, the mean HBR of all *Anopheles* was 61.3 bpn in N’Gattakro and 15.4 bpn in Odiennekourani (Table 5). The difference was significant (w = 74; *p* = *0.0019*). Among the 1766 *Anopheles* specimens analysed by qPCR, 12 were infected by *Plasmodium* parasites (n = 9 *An. coluzzii* and n = 3 *An. gambiae s.s.*); this gave an overall infection rate of 0.68%. Two *Plasmodium* species were identified: *Plasmodium falciparum* (n = 8 *An. coluzzii*; 0.71% and n = 3 *An. gambiae s.s.;* 0.5%) and *Plasmodium malariae* (n = 1 *An. coluzzii*; 0.09%).

**Table 5:** Entomological parameters of the *Anopheles* specimens infected by *Plasmodium* parasites at the two lowlands in function of the season

Lowland	Season	No. positive	No. tested	IR (%) [IC 95%]	HBR	EIR
					(bpn)	(ibpn)
N’Gattakro	Rainy season	7	835	0.84 [0.34–1.72]	60.2	0.505
	Dry season	4	415	0.96 [0.26–2.45]	62.8	0.604
Total		11	1250	0.88 [0.44–1.57]	61.3	0.539
Odiennekourani	Rainy season	1	293	0.34 [0.008–1.88]	16.1	0.055
	Dry season	0	223	0 [0.00–1.64]	14.3	0
Total		1	516	0.19 [0.005–1.07]	15.40	0.029

*No.* number of mosquitoes

Among the 12 infected mosquitoes, 11 were captured in N'Gattakro (n = 7 in the rainy season and n = 4 in the dry season) and 1 in Odiennekourani (rainy season) (Table 5). However, there is no relationship between the number of infected mosquitoes and the areas (OR = 4.57; IC95% = [0.66; 197.03]; *p* = *0.19*). Eight infected specimens were captured outdoor and four indoor. Seven infected anopheles were captured before midnight in N’Gattakro, and the infected specimen in Odiennekourani was collected between 03:00 and 04:00 am.

The entomological inoculation rate was higher in N’Gattakro (0.539 ibpn) than in Odiennekourani (0.029 ibpn) (w = 69; *p* = *0.0059*). It was 0 ibpn in the dry season in Odiennekourani.

## Discussion

The study was designed to assess malaria transmission in the surroundings of two lowlands under agricultural development in the city of Bouaké. Some differences were observed in the entomological drivers of malaria transmission in the two studied lowlands despite the same cultivations.

*Anopheles gambiae s.l.* predominated in N’Gattakro and *Cx. quinquefasciatus* in Odiennekourani. The high abundance of *Cx. quinquefasciatus* in Odiennekourani could be associated with the permanent wastewater stream that favours its development, as observed in urban areas in Benin by Salako et al. [31]. The organic pollution of the watercourse that irrigates the rice plots and to the hand-watering of the market-gardening plots, could also explain the lower *An. gambiae s.l.* abundance in Odiennekourani than in N'Gattakro. Conversely, the higher water quality and its permanence for irrigation might explain its abundance in N’Gattakro. The *An. gambiae s.l.* preference for non-polluted water is generally accepted [32]. However, some studies showed that this species can be found also in polluted water [33, 34], although the pollution type and level have been rarely measured.

Overall, four *Anopheles* species were identified: *An. gambiae s.l.* and *An. funestus s.l.* are considered the main vectors of *Plasmodium sp*. in Côte d’Ivoire, while *An. pharoensis* and *An. ziemanni* appear as potential vectors. *Anopheles coustani,* which had been observed by Dossou-Yovo et al. [17], was not found. This could be due to the ongoing urbanization that might have altered the vector composition [35]. The dramatic increase in human densities in urban areas could lead to a shift in the feeding choices of *Anopheles* towards humans in many cities in sub-Saharan Africa. [36]. Unlike *An. arabiensis* which is highly flexible in its host preferences, *An. coustani* is a zoophilic species that may not have been able to adapt to the scarcity of animal hosts linked to the urban lifestyle. Furthermore, *An. coustani* may have failed to adapt to water pollution usually associated with urban development as opposed to *An. gambiae s.l*. [34].

Molecular analysis revealed the presence of *An. gambiae s.s.* and *An. coluzzii,* as previously reported in the region of Bouaké [11, 37]. *Anopheles coluzzii* was more abundant than *An. gambiae s.s.* in N’Gattakro. *Anopheles coluzzii* shows some adaptation to the urban environment and can colonize lowland areas where it might find more or less permanent larval habitats, unlike *An. gambiae s.s.* that prefers small and temporary sites [3, 38, 39]. Capturing *Anopheles* all over the district, not only along the lowlands, Adja et al. [11] identified more *An. gambiae s.s.* than *An. coluzzii* in N'Gattakro. The existence of more favourable larval habitats for this species within the district can be presumed.

Molecular analysis also resulted in the identification of *An. arabiensis* in Odiennekourani only. Its presence was recently reported for the first time in Côte d’Ivoire [40]. *Anopheles arabiensis* is generally associated with arid environments [41]. However, urbanization can provide favourable conditions for its development, as observed in Ghana, Nigeria [42]. The development of *An. arabiensis* in urban environments is consistent with its tolerance for polluted aquatic habitats as it was observed in different cities as Dakar [43] and Ouagadougou [44]. *Anopheles arabiensis* low abundance in Bouaké suggests that this species is currently in the process of being introduced in the country, possibly from Burkina Faso. Indeed, *An. arabiensis* is now prevalent in Bobo-Dioulasso (500 km from Bouaké) [45], and its transport via the intense commercial exchanges between these cities, cannot be excluded (e.g. with livestock carried by truck). Eritja et al. [46] demonstrated the importance of passive transportation in cars in *Aedes albopictus* colonization in Spain. This hypothesis needs to be tested by comparing the populations of Bouaké and Bobo-Dioulasso.

In N’Gattakro, *An. gambiae* complex species showed a trend towards exophagy that was not observed in Odiennekourani. From 2014 to 2015, Adja et al. [11] did not observe this trend in N’Gattakro. Conversely, in 2020, Assouho et al. [47] showed that all malaria vectors were frequently caught outdoors in urban and rural areas in different Côte d’Ivoire areas. Behavioural changes have been documented amongst *Anopheles* following the use of insecticide-treated nets (ITNs) and indoor residual spraying (IRS), including outdoor biting, time of biting and host choice preference [48–50]. As a limitation of the study, LLINs use among the human populations of both districts was not surveyed. The only hypothesis that can be formulated is that people in N’Gattakro are more likely to use LLINs than those in Odiennekourani, thus leading malaria vectors in N’Gattakro to be more exophagic.

Together with the presence of *An. arabiensis* (an exophilic and exophagic vector [51]) and the noticeable percentage of outdoor bites (70.9%) occurring when people are not supposed to be under bed nets in both lowlands, these results highlight the risk of residual transmission that could compromise malaria control. However, the risk of human population exposure to malaria vectors requires further investigation regarding the use of mosquito nets, the time of sleeping and of waking up [52].

People in N’Gattakro received 15-fold more anopheles bites than those in Odiennekourani, and they would annually suffer about 200 infected bites per human compared to about 10 in Odiennekourani. Such differences within a same area was already recorded [53]. These authors reported an average EIR around 300 infected bites per human and per year in Côte d’Ivoire, highlighting that the EIR in N’Gattakro is quite high for an urban area. Even if the association between the EIRs and the intensity of transmission still remains difficult to interpret, it can be considered that a greater risk of transmission occurred in N’Gattakro where the surface area of rice fields is similar to that of Odiennekourani but market gardening is less extensive.

Irrigated rice fields are known to produce important numbers of *Anopheles*, although a few of these are infected [54]. Shah et al. [55] recently showed that in complex mosaic cropping systems interspersed with natural vegetation, the risk of malaria increased in urban areas. These results prompt a further study of larval habitat production over time at the crop plot scale. The lowlands under study consist of crop mosaics that may have varying effects on *Anopheles* larvae production. Investigating them at a fine scale may allow to better understand larval production and design less productive crop assemblages, notably by introducing shade trees as suggested by Dongus et al. [56].

## Conclusion

The results highlight the existence of uneven malaria transmission risk in the two surveyed lowlands, possibly associated with lowland management practices that should be thoroughly investigated, especially the management of parcels watering. They also indicate that living close to an agriculturally developed lowland may increase the risk of exposure to malaria vector bites. Given the importance of urban agriculture in Bouaké, *Anopheles* exophagic behaviour and the proportion of bites occurring when people are not supposed to be under mosquito nets, these results suggest a possible risk of residual transmission that could hamper the malaria control efforts through the use of long-lasting insecticidal nets. Several research topics could be explored to help the National Malaria Control Programme, including trials of additional control tools for LLINs, such as eave tubes, surveys to better understand population behaviour in terms of LLIN use, and also studies on developing lowland agriculture that would produce less *Anopheles*.

## Data Availability

The datasets used and/or analysed in the current study are available from the corresponding author on reasonable request.
